# Correlation between the Serum Platelet-Derived Growth Factor, Angiopoietin-1, and Severity of Coronary Heart Disease

**DOI:** 10.1155/2020/3602608

**Published:** 2020-09-10

**Authors:** Sisi Pang, Zhengxian Tao, Xiaoyan Min, Chuanwei Zhou, Dijia Pan, Zhen Cao, Xiangming Wang

**Affiliations:** ^1^Department of Geriatric Cardiology, The First Affiliated Hospital of Nanjing Medical University, Nanjing, China; ^2^Department of Cardiology, The First Affiliated Hospital of Nanjing Medical University, Nanjing, China

## Abstract

**Background:**

The expression of the platelet-derived growth factor (PDGF), angiopoietin-1 (Ang-1) in patients with coronary artery disease of different studies was inconsistent. This study was to investigate the expression of the PDGF and Ang-1 in peripheral blood and coronary artery in patients with acute coronary syndrome (ACS) and the relationship between the expression of the PDGF and Ang-1 and the severity of coronary artery disease.

**Methods:**

A total of 81 patients with acute coronary syndrome undergoing coronary angiography were enrolled from September 2012 to December 2013. Patients with ACS included 61 patients with acute myocardial infarction (AMI group) and 20 patients with unstable angina pectoris (UAP group). The 29 patients who were hospitalized for chest pain undergoing coronary angiography without stenosis and with TIMI level 3 blood flow were selected as the control group. During coronary arteriography (CAG) or percutaneous coronary intervention (PCI), blood in the peripheral artery and in the local coronary artery was collected from all the patients. Serum PDGF and Ang-1 levels were measured by ELISA. We calculated the Gensini score of each patient with CHD according to the result of CAG. Patients with ACS were followed up, and the major adverse cardiovascular and cerebrovascular adverse events were recorded.

**Results:**

In peripheral blood, the concentration of the PDGF was significantly elevated in the ACS group than that of the control group. The level of the PDGF in the AMI group was significantly higher than that in the UAP group. In coronary artery blood, the level of the PDGF in the ACS group was significantly higher than that of the UAP group. There was no significant difference in the concentration of Ang-1 in peripheral blood between patients with coronary heart disease and the control group. The concentration of Ang-1 in the coronary artery was significantly lower than that in peripheral blood. The coronary Ang-1 concentrations in the ACS group were significantly higher than those in the UAP group. The concentrations of the PDGF and Ang-1 in peripheral and coronary artery blood were positively correlated with the severity of coronary lesions. Patients with MACCE had higher PDGF and Ang-1 levels in the coronary sinus.

**Conclusion:**

The serum PDGF concentration in patients with acute coronary syndrome was significantly increased, especially in the local coronary artery. The serum Ang-1 in the coronary artery was significantly increased in patients with acute myocardial infarction and was related to the degree of coronary artery stenosis. Coronary sinus PDGF and Ang-1 levels can reflect the severity of lesions in patients with acute coronary syndrome.

## 1. Introduction

Acute coronary syndrome (ACS) is a type of clinical critical illness of coronary heart disease (CHD) that seriously endangers human health. A large number of cytokines secreted by cells participate in the pathogenesis of ACS through different pathophysiological pathways. Several studies have shown that the platelet-derived growth factor and angiopoietin play an important role in vascular endothelial injury and angiogenesis during ACS pathology.

The platelet-derived growth factor (PDGF) is mainly derived from platelets and also exists in damaged endothelial cells, fibroblasts, smooth muscle cells, macrophages, and mesangial cells [1]. The current study suggests that the PDGF binding to its receptor exerts biological effects and participates in various pathophysiological development processes. PDGF-BB participates in the development of atherosclerosis through chemotaxis, the mitogenic effect, and vasoconstriction and can promote neovascularization, which plays an important role in various tissue damage repair and disease processes [2]. Angiopoietin is an angiogenic growth factor secreted by the vascular endothelium and a ligand for the tyrosine kinase receptor Tie2. A number of studies have confirmed that Ang-1 has the function of maintaining the maturation and stability of vascular endothelial cells and promoting budding and branching during angiogenesis [3]. Ang-1 and Ang-2 play an important role in the establishment of angiogenesis and collateral circulation in ischemic heart disease.

However, the relationship between plasma PDGF, Ang-1 concentration, and severity of coronary artery disease in patients with different types of coronary heart disease remains controversial. In addition, the same cytokine has different functions in different internal environments. Previous studies have focused on the role of cytokines in the peripheral circulation. This study investigated the differential expression of serum PDGF and Ang-1 in peripheral blood and coronary artery in patients with different types of coronary heart disease and its relationship with the severity of coronary artery disease.

## 2. Materials and Methods

### 2.1. Study Population

A total of 81 patients with acute coronary syndrome undergoing coronary angiography (CAG) were enrolled from the department of cardiology in the first affiliated hospital of Nanjing Medical University from September 2012 to December 2013. Patients were divided into different groups based on coronary angiography and clinical symptoms. Exclusion criteria: (1) patients with severe heart failure (LVEF ≤ 30%), (2) severe cardiomyopathy and pericardial disease, (3) acute stroke, (4) severe complications (liver failure, renal failure, connective tissue disease, cancer, and infectious diseases), (5) acute hemorrhagic diseases, and (6) severe allergies. Eighty-one patients with ACS were enrolled, including 61 patients with acute myocardial infarction (AMI) and 20 patients with unstable angina pectoris (UAP). There were 50 males and 11 females, aged 31–84 (60.4 ± 11.5) years in the AMI group and 20 cases of UAP which had 14 males and 6 females aged 49–80 (62.2 ± 8.5) years. In addition, patients who underwent coronary angiography due to chest pain without coronary stenosis and with TIMI level 3 blood flow were selected as the control group (non-CHD group, control). In the control group, there were 16 males and 13 females, aged 45–84 (59.6 ± 9.6) years. Acute coronary syndrome includes acute myocardial infarction and unstable angina pectoris. The clinical diagnosis of AMI was based on the concurrence of prolonged (>30 minutes) chest pain or discomfort, elevated myocardial enzymes (raised troponin T levels to, at least, the high-risk level), and ECG changes (ST-T segment elevation or depression). Unstable angina pectoris (UAP) is defined as the presence of typical angina at rest or on minimum exertion associated with acute and transient ST-T segment ECG changes but with normal cardiac enzymes. All patients received standard therapy, including aspirin, low-molecular-weight heparin, intravenous nitrates, a statin, *β*-blocker, and ACE inhibitor, when appropriate.

### 2.2. Clinical Characteristics and Biochemical Indicators

Collection of the basic characteristics of all subjects including gender, age, body mass index (BMI), previous histories of disease (hypertension, diabetes, stroke, hyperlipidemia, and other previous medical history), and smoking was performed. Clinical examination (systolic blood pressure, diastolic blood pressure, and heart rate) was also performed. The biochemical indicators including total cholesterol (CHOL), low-density lipoprotein (LDL-C), high-density lipoprotein (HDL-C), triglyceride (TG), serum creatinine, uric acid, liver function, and serum glucose were determined using an automate biochemistry analyzer (Roche Diagnostic).

### 2.3. Coronary Angiography

Before CAG, each patient underwent radial artery or femoral artery puncture and insertion of the artery sheath. Patients were examined by CAG with a GE Innova3000 angiography machine. The JL and JR contrast catheters or appropriate catheters were selected and placed into the left and right coronary arteries, respectively. The multiposition projection was used, and the coronary angiography result was recorded. Noncoronary heart disease is defined as the normal or narrowed lumen diameter of contrast vessels <50%; coronary artery disease is defined as a major vascular stenosis greater than 50%.

### 2.4. Severity Determination of Coronary Artery Disease

We used the Gensini scoring system [4] to evaluate the severity of CAD. Two professional physicians who had long been involved in coronary intervention evaluated the angiographic findings according to the location of the lesion. Stenosis in the coronary artery of <25%, 26–50%, 51–75%, 76–90%, 91–99%, and 100% was scored as 1, 2, 4, 8, 16, and 32 point, respectively. 1–30 score was divided into the low-risk group, 30–60 was divided into the medium risk group, and a score of ≧60 was divided into the high-risk group.

### 2.5. Determination of PDGF and Ang-1 Levels

Peripheral artery blood and coronary blood were drawn from patients at the time of the CAG or PCI procedure. 5 ml artery blood (peripheral blood) was drawn out from the radial artery or the femoral artery before coronary angiography and after the successful implantation of arterial sheath puncture. In patients with ACS, a 6-F JR angiographic catheter was also used for the collection of coronary sinus blood. All blood was centrifuged at 3500 r/min for 15 min at room temperature, and then, 0.5 ml serum was taken and kept frozen at −70°C. Serum levels of the PDGF and Ang-1 were measured using the PDGF-BB, Ang-1 ELISA kit (DVE00; R&D systems; Abingdon, Oxon, UK) following the manufacturer's instructions.

### 2.6. Follow-Up of Outcomes

Patients with ACS were followed up by telephone once a month, and outpatient visits were conducted every three months. Adverse events were recorded prospectively during follow-up. The last follow-up date for all participants was December 2018. The primary outcome was recurrent major cardiovascular and cerebrovascular adverse events (MACCE), defined as cardiac death, recurrent ACS, unplanned revascularization, heart failure, and stroke. Cardiac death was defined as death from cardiovascular cause or unexpected death within 24 h of symptom onset. Recurrent ACS was the composite of ST elevation myocardial infarction (MI), non-ST elevation MI, and unstable angina. AMI was defined by ICD 10 codes I21 and I22 and unstable angina by code I20. Heart failure was diagnosed for patients with clinical symptoms including dyspnea, shortness of breath, and peripheral edema together with evidence of heart failure by Pro-BNP or echocardiography examination. Stroke was defined as patients with a newly developed neurological deficit and relevant findings on computed tomography or magnetic resonance imaging.

### 2.7. Statistical Analysis

Data analysis was performed using SPSS 21.0 software. The measurement data were expressed as X ® ± s, the *t*-test was used for the comparison between groups, and ANOVA analysis was used to compare the three groups; the count data were expressed as frequency, and the *χ* [2] test was used to compare the two groups. ACS patients with coronary artery and peripheral blood concentrations of growth factors were compared with the paired *T*-test, and ACS patients with peripheral blood and the control group were compared using one-way ANOVA. Pearson correlation analysis was used to analyze the relationship between the single factor and coronary Gensini score. Kaplan–Meier curves were used to analyze the risk of the 36-month MACCE follow-up among different groups. *P* < 0.05 was considered a statistically significant difference.

## 3. Results

### 3.1. Baseline Characteristics of the Patients

The age, smoking history, fasting blood glucose, and LDL-C levels in the ACS group of coronary heart disease were significantly different compared with the control group (*P* < 0.05). In the ACS group, compared with the unstable angina group, the proportion of hypertension, smoking, and LDL-C levels in patients with acute myocardial infarction was significantly increased (*P* < 0.05). There was no significant difference in other indicators among the three groups (*P* > 0.05) (Shown in Table 1).

### 3.2. Serum PDGF Levels in Each Group of Patients

Compared with the control group, the concentration of the PDGF in peripheral blood of the ACS group was significantly higher than that of the control group (*P* < 0.05). The level of the PDGF in the AMI group was significantly higher than that in the UAP group (*P* < 0.05). In the ACS group, the concentration of the PDGF in coronary sinus blood was higher than that in peripheral blood (*P* < 0.01). Compared with the UAP group, the concentration of the PDGF in coronary sinus blood of the ACS group was significantly higher than that of the UAP group (*P* < 0.01) (Shown in Table 2).

### 3.3. Serum Ang-1 Levels in Each Group of Patients

There was no significant difference in the concentration of Ang-1 in peripheral blood between patients with coronary heart disease and control group (*P* > 0.05). There was no significant difference in the peripheral blood Ang-1 concentration between AMI patients and UAP patients (*P* > 0.05). In the ACS group, the concentration of Ang-1 in coronary sinus blood was significantly lower than that in peripheral blood (*P* < 0.01), and coronary Ang-1 concentrations in the ACS group were significantly higher than those in the UAP group (*P* < 0.01) (Shown in Table 2).

### 3.4. Relationship between the Plasma PDGF, Ang-1 Level, and Gensini Score

**Figure 1:**
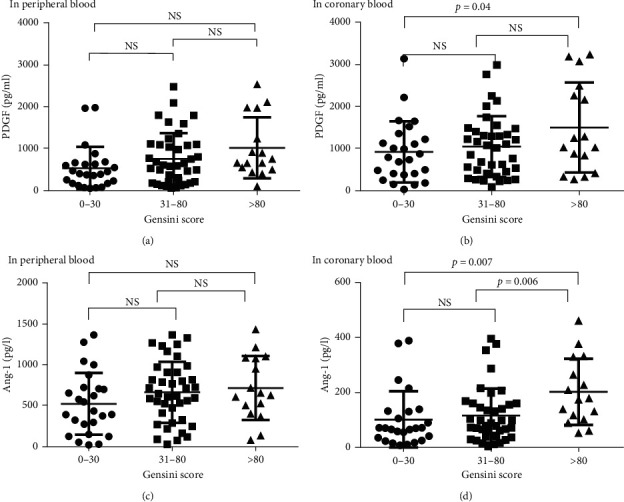
Peripheral blood PDGF and Ang-1 concentrations in different Gensini score groups.

According to the Gensini score, all patients with ACS were divided into a low-risk group (1–30 points), medium-risk group (30–80 points), and high-risk group (≧80 points). PDGF levels in peripheral blood showed an upward trend from the low-risk group to high-risk group, but there was no significant difference between the three groups (*P* > 0.05). There were statistical differences of PDGF concentrations in the local coronary artery between the three groups. The levels of the PDGF in the peripheral blood of the high-risk group were significantly higher than those in the low-risk group (*P* < 0.05), but there was no significant difference between the high-risk group and the moderate-risk group (Shown in Figure 1(a)). The high-risk group of patients had the coronary artery PDGF concentration significantly lower than that of the low-risk group (*P* < 0.05), and no significant difference was found with the medium-risk group (shown in Figure 1(b)). There was no significant difference of the PDGF concentration in peripheral blood and the PDGF concentration in the coronary artery between the medium-risk group and low-risk group.

As shown in Figures 1(c)–1(d), there was no significant difference in the concentration of Ang-1 in peripheral blood between the ACS group and low-risk group and medium-risk group and high-risk group (*P* > 0.05). The concentration of Ang-1 in the coronary heart disease group was higher than that of the low-risk group, medium-risk group, and high-risk group, and the difference was statistically significant (*P* < 0.01). In the high-risk group, the concentration of Ang-1 in the coronary artery was significantly lower in the low-risk group than in the medium-risk group (*P* < 0.05). There was no significant difference in the concentrations of Ang-1 in the medium-risk group and the low-risk group.

**Figure 2:**
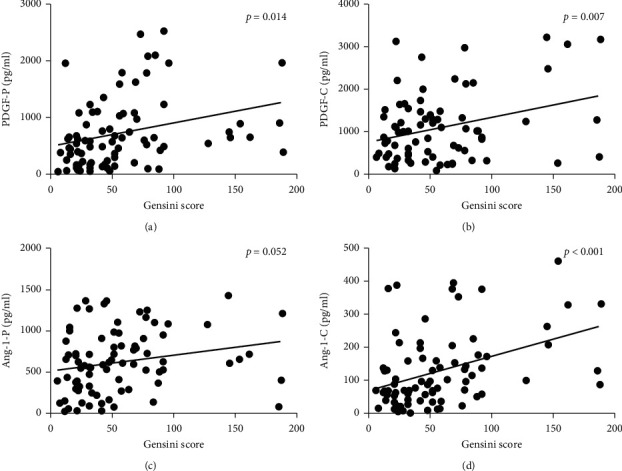
Relation of peripheral blood PDGF and Ang-1 levels with the Gensini score. (a) Relation of the peripheral blood PDGF level with the Gensini score. (b) Relation of the coronary blood PDGF level with the Gensini score. (c) Relation of the peripheral blood Ang-1 level with the Gensini score. (d) Relation of the coronary blood Ang-1 level with the Gensini score.

Person correlation analysis showed that the PDGF concentration in peripheral blood was mildly positively correlated with the Gensini score (rho = 0.273, *P*=0.0014). Similarly, the levels of the PDGF in the local coronary artery were also positively correlated with the severity of coronary lesions (rho = 0.240, *P*=0.03) (see Figures 2(a) and 2(b)). Pearson-related analysis showed a positive correlation between the concentration of Ang-1 in peripheral blood and the degree of coronary artery stenosis (rho = 0.217, *P*=0.052), while there was a positive correlation between the concentration of Ang-1 in coronary artery and the severity of coronary artery disease (rho = 0.399, *P* < 0.001) (see Figures 2(c)–2(d)).

### 3.5. Relationship between the Plasma PDGF, Ang-1 Level, and MACCE during the 36-Month Follow-Up

**Figure 3:**
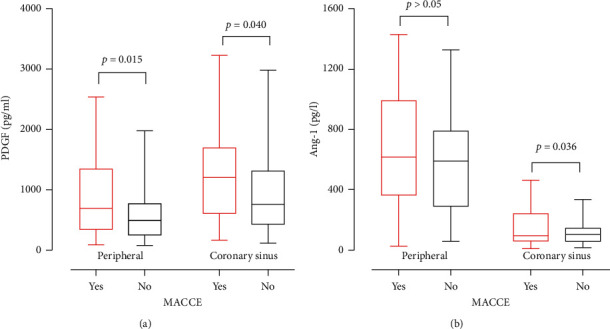
Relation of the PDGF and Ang-1 with (yes) and without (no) incident MACCE in 3 years. (a) Box–Whisker plots of PDGF levels among acute coronary syndrome patients depicting the relation of the PDGF with (yes) and without (no) incident MACCE in 3 years. (b) Box–Whisker plots of Ang-1 levels among acute coronary syndrome patients depicting the relation of PDGF with (yes) and without (no) incident MACCE in 3 years.

**Figure 4:**
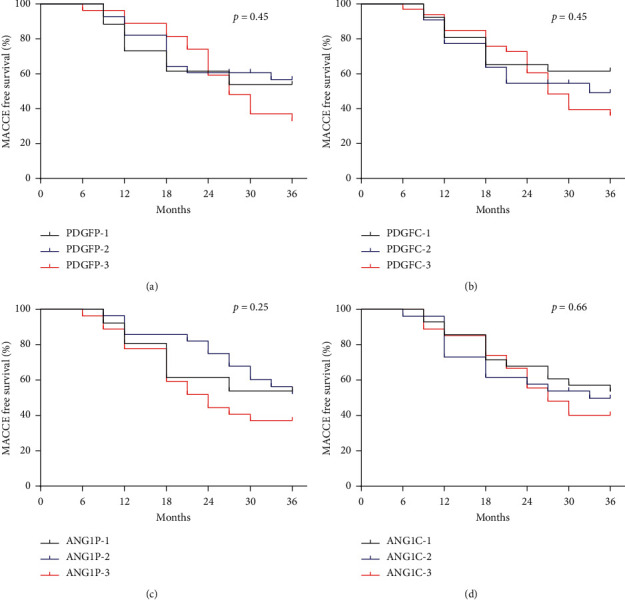
Kaplan–Meier curves of 3-year major adverse cardiac and cerebrovascular events risk stratified by the tertiles of the cellular factor level. ((a) PDGF in peripheral blood; (b) PDGF in the coronary sinus; (c) Ang-1 in peripheral blood, and (d) Ang-1 in the coronary sinus).

The average PDGF levels in peripheral blood were higher in patients with MACCE than in patients without MACCE (*P*=0.015, Figure 3(a)). Patients with MACCE had higher PDGF level in the coronary sinus than patients without MACCE (*P*=0.04, Figure 3(a)). The average Ang-1 level in the coronary sinus was higher among patients with MACCE, at least, in the 36-month follow-up period than that without MACCE (*P*=0.036, Figure 3(b)). All ACS patients were divided into three groups according to the tertiles of the PDGF level and Ang-1 level. Kaplan–Meier curves for 36-month MACCE illustrated that the risk of MACCE was not different among the three groups stratified by the tertiles of the PDGF in peripheral blood (*P* > 0.05, Figure 4(a)), the PDGF in the coronary sinus (*P* > 0.05, Figure 4(b)), Ang-1 in peripheral blood (*P* > 0.05, Figure 4(c)), and Ang-1 in the coronary sinus (*P* > 0.05, Figure 4(d)).

## 4. Discussion

In this study, we observed differences in serum PDGF and Ang-1 concentrations in peripheral and coronary arteries of patients with different types of coronary heart disease. We found that (1) the serum PDGF concentration in patients with acute coronary syndrome was significantly increased, especially in the local coronary artery, and it was positively correlated with the severity of coronary artery disease; (2) the concentration of Ang-1 in the peripheral blood of the early stage of ACS did not change significantly, but the serum Ang-1 in the coronary artery was significantly increased in patients with acute myocardial infarction and was related to the degree of coronary artery stenosis; and (3) patients with MACCE had higher PDGF and Ang-1 levels in the coronary sinus.

Acute coronary syndrome is a type of clinical critical illness of coronary heart disease that seriously harms human health. The main pathogenesis of ACS includes endothelial dysfunction, coronary plaque formation, and rupture, resulting in platelet aggregation and thrombosis in the coronary vascular lumen [5]. In addition, coronary microcirculation dysfunction also plays an important role in the pathological mechanism of ischemic heart disease [6, 7]. Many studies have confirmed that several cytokines secreted by cells participate in the pathogenesis of ACS through different pathophysiological pathways. Studies have shown that the platelet-derived growth factor and angiopoietin play an important role in vascular endothelial injury and angiogenesis in the pathogenesis of ACS [2]. However, there are still controversies about the relationship between plasma PDGF and Ang-1 levels and the severity of coronary artery disease in different types of patients with coronary heart disease. In addition, the same cytokine may have different functions in different internal environments. Previous studies have focused on the role of cytokines in the peripheral circulation; however, the difference in the local physiological function of coronary circulation in ACS patients is still unclear.

The platelet-derived growth factor is a 24 Ku cation glycoprotein, which is mainly derived from platelets, and also present in injured endothelial cells, transitional fibroblasts, smooth muscle cells, macrophages, and mesangial cells [1]. There is still a discussion on whether the relationship between the plasma PDGF concentration and severity of coronary artery disease in different types of coronary heart disease patients still exists [8]. Nakagawa et al. [9] showed that high expression of PDGF-BB mRNA was detected in coronary lesions in patients with ACS. Similarly, Koizumi et al. [10] found that PDGF-BB levels in infarct-related vessels were significantly higher in patients with ST-segment elevation myocardial infarction (STEMI) than in non-infarct-related vessels, and this high level was maintained up to 48 h after surgery. All of the abovementioned results suggest that high levels of PDGF-BB are associated with coronary plaques.

In this study, serum PDGF concentrations in peripheral blood and coronary arteries of patients with acute coronary syndrome were measured and compared with the non-coronary heart disease group. The results showed that the serum PDGF concentration in peripheral blood of patients with ACS was significantly higher than that in the non-coronary heart disease group. The results may be related to the following mechanisms: firstly, after the intimal injury, platelets are activated, which adhere to and aggregate in the injured area and release PDGF-BB, in order to induce the migration and proliferation of mesenchymal smooth muscle cells to the intima. Secondly, cell function is activated and thrombin is produced, which can activate a series of reactions of coagulation cascade and stimulate platelet release PDGF-BB. Thirdly, in ACS patients, local hemodynamics of diseased vessels is changed and the production of PDGF-BB by platelets and endothelial cells subjected to shear force increases. In summary, platelet aggregation activation occurs immediately after vascular injury in the injured area, and activated platelets and endothelial cells release a large number of platelet-derived growth factors. Our study also found that the serum PDGF concentration in patients with acute myocardial infarction, whether in peripheral blood or coronary artery, was significantly higher than that in patients with unstable angina. We think the reason is that platelet activation and inflammation in the pathological process of AMI were more intense than UAP. Most of the patients with AMI had coronary artery occlusion or subtotal occlusion with heavy thrombosis load, and platelet stress reaction maybe correspondingly larger. This study was the first to observe the changes of local PDGF in coronary artery in patients with ACS. The study found that the local concentration of PDGF in ACS patients was significantly higher than that in peripheral blood, which further reflected the significant increase of platelet activation in coronary artery in patients with ACS. On the one hand, instability of coronary plaque in ACS patients leads to plaque rupture, platelet activation, and release of more platelet-derived factors in the coronary arteries. On the other hand, inflammation plays a significant role in the progression of atherosclerotic disease. The inflammatory response in ischemic myocardial tissue also stimulates the local release of cytokines such as inflammatory factors and the PDGF, further increasing the local PDGF concentration in the coronary arteries.

Angiopoietin is an angiogenic growth factor secreted by the vascular endothelium and is a ligand for the tyrosine kinase receptor Tie2. Angiopoietin-1 is mainly expressed in the embryonic vascular stroma and adult lung, coronary artery, skin, and other tissues [11]. In the regulation of angiogenesis, angiopoietin-1 does not directly regulate endothelial cell proliferation, but binds to the Tie2 receptor, promotes receptor phosphorylation, and regulates vascular maturation of blood vessels from the endothelial cell layer into a multicellular fine vascular structure. The process and maintenance of vascular structures is through cell-cell and cell-matrix interactions [12,13]. Different studies have found changes in angiopoietin in different types of coronary heart disease [14,15]. Lee et al. [16] found that, after induction of myocardial ischemia, the expression of the vascular endothelial growth factor (VEGF) and angiopoietin-2 in myocardial tissue significantly increased, reaching a peak on the third day of ischemia and, then, decreased, but Ang-1 remained unchanged. Liu et al. [17] found that decreased plasma Ang-1 levels on admission, left ventricular ejection fraction, and multivessel disease independently predicted the development of 1-year MACCEs in patients with st-segment elevation myocardial infarction (STEMI). Plasma levels of Ang-1from acute to chronic phase in patients with acute myocardial infarction and sequence changes in their interaction may reflect a gradual progression of angiogenesis after myocardial ischemic necrosis development process. Our study found that the concentration of Ang-1 in peripheral blood of patients with coronary heart disease was not significantly different from that of the control group. In the ACS group, the concentration of Ang-1 in the coronary arteries was significantly lower than that in the peripheral blood. This result is basically consistent with the results of previous international studies. The reason for this may be that the release of Ang-1 in peripheral blood is not obvious in the acute phase of ACS. Ang-1 may play a role mainly in the process of vascular remodeling and maturation in the late stage of angiogenesis and is closely related to the involvement of the VEGF.

During an acute coronary event, angiopoietin production increases and gradually accumulates in the coronary arteries, promoting further angiogenesis. The study found that the serum Ang-1 concentration in the coronary artery of patients with AMI was higher than that of UP; the presumed reason may be as follows: (1) the inflammatory response of the coronary artery in patients with AMI is more intense than that in patients with unstable angina, and the local serum Ang-1 concentration is, therefore, high. (2) In UP, unstable angina myocardial tissue is not necrotic, and the formation of angiogenic material and local aggregation of coronary artery is not as significant as AMI. Local myocardial necrosis in patients with AMI leads to the release of angiogenic substances and gradually accumulates in the coronary artery. Therefore, the concentration of Ang-1 in peripheral blood of patients with AMI was not statistically different from that of the control group. We speculate that the role of Ang-1 is mainly concentrated in the stable phase of AMI. More Ang-1 will accumulate in the coronary artery and cooperate with the VEGF to reshape the new blood vessels, increase the diameter of the tube, and increase the branch to promote it, mature, can also promote the connection between endothelial cells and endothelial cells and extracellular matrix, maintain the normal structure and stability of new blood vessels, and further participate in advanced collateral angiogenesis.

The study further explored the correlation between the serum PDGF, Ang-1 concentration, and severity of coronary artery disease. Correlation analysis showed that the serum PDGF concentration in the peripheral blood and coronary artery was positively correlated with the Gensini score, and it was found that, with the increase of the serum PDGF concentration, the patient's Gensini score gradually increased, and the average Gensini score of the high concentration group was significantly higher than that of the low concentration group. High Gensini scores indicate a high severity of coronary artery disease, and the incidence of plaque rupture, platelet activation, and thrombotic events is higher. We also found that it is positively correlated between local serum Ang-1 and the severity of coronary artery disease. Patients with high Gensini scores often have multivessel disease, calcified lesions, and diffuse coronary lesions [18]. Therefore, patients with severe coronary artery disease will stimulate the increase of local angiogenic material in the coronary arteries, which is manifested as an increase in the local Ang-1 level of the coronary artery. The more severe the lesion, the more prominent the accumulation of angiogenin in the coronary artery. Therefore, the study suggests that the serum PDGF and Ang-1 concentration in the coronary artery can be used as an important indicator to determine the severity of vascular disease and the degree of inflammatory response in patients with coronary heart disease. Identification increases the reference value.

## 5. Additional Points

First, our present study was a cross-sectional single-center study with low participants' number. The clinical application of these findings will require further validation of large multicenter studies and a larger number of patients for reliable evaluation. Second, in this study, we measured the plasma PDGF and Ang-1 levels at only one time. Further studies are needed to measure the different time points for the PDGF and Ang-1 to predict the prognosis. Third, the PDGF has several subtypes, and the potential different role of PDGF subtypes remains uncertain. Therefore, different plasma PDGF forms could be measured to observe among different groups. Fourth, the Gensini score relies on a visual evaluation of coronary angiography with existing interobserver variability.

## 6. Conclusions

We showed that serum PDGF concentrations in peripheral blood and coronary arteries can reflect the extent of platelet activation and inflammatory response in coronary artery lesions in acute coronary syndrome. Coronary sinus Ang-1 levels can reflect the severity of lesions in patients with acute myocardial infarction. The PDGF and Ang-1 can be used as indicators of the severity and outcome of patient with acute coronary syndrome.

## Figures and Tables

**Table 1:** Baseline characteristics of all the study subjects.

Variables	Control (*n* = 29)	ACS groups		
		Total (*n* = 81)	UAP groups (*n* = 20)	AMI groups (*n* = 61)
Age (years)	59.62 ± 9.59	60.84 ± 0.78^*∗*^	62.2 ± 8.46^*∗*^	60.39 ± 11.46^*∗*^
Female, [*n* (％)]	16 (55.17%)	64 (79.01%)	14 (70.00%)	50 (81.96%)
Hypertension, [*n* (％)]	20 (68.97%)	49 (60.49%)	9 (45%)	40 (65.57%)^#^
Diabetes, [*n* (％)]	11 (37.93%)	14 (17.28%)	4 (20%)	10 (16.39%)
Smoking, [*n* (％)]	4 (13.79%)	40 (49.38%)^*∗*^	6 (30%)^*∗*^	34 (55.74%)^*∗#*^
BMI (kg/m3)	23.56 ± 2.69	24.86 ± 3.14	23.37 ± 2.57	25.34 ± 3.17

History of medication				
Nitrates, [*n* (％)]	13 (44.83)	47 (58.02)	16 (80.0)	31 (50.82)
Beta blockers, [*n* (％)]	10 (34.45)	41 (50.62)	9 (45.0)	32 (52.46)
ACEIs/ARBs, [*n* (％)]	12 (41.38)	33 (40.74)	7 (35.0)	26 (42.62)
CCBs, [*n* (％)]	9 (31.03)	12 (14.81)	5 (25.0)	7 (11.47)
Aspirin, [*n* (％)]	29 (100)	81 (100)	20 (100)	61 (100)
Clopidogrel, [*n* (％)]	16 (55.17)	80 (98.77)^*∗*^	20 (100)^*∗*^	60 (98.36)^*∗*^
SBP (mmHg)	133.45 ± 15.68	128.64 ± 20.52	133.85 ± 17.71	126.93 ± 21.22
DBP (mmHg)	80.03 ± 7.62	78.42 ± 12.49	78.55 ± 9.08	78.38 ± 13.49
FBS (mmol/l)	5.58 ± 1.30	6.83 ± 2.60^*∗*^	5.87 ± 2.12	7.15 ± 2.68^*∗#*^
HbA1c (%)	6.58 ± 1.45	6.65 ± 2.16	6.01 ± 0.81	6.86 ± 2.42
TC (mmol/l)	4.57 ± 1.07	4.97 ± 1.33	4.43 ± 1.17	4.52 ± 1.22
LDL-C (mmol/l)	2.71 ± 0.77	2.88 ± 0.94^*∗*^	2.61 ± 0.76	2.97 ± 0.98^*∗*^
HDL-C (mmol/l)	1.21 ± 0.27	1.08 ± 0.65	1.12 ± 0.34	1.06 ± 0.33
TG (mmol/l)	1.78 ± 1.03	1.69 ± 0.1.22	1.46 ± 0.71	1.76 ± 1.35
SUA (*μ*mol/l)	319.87 ± 80.32	318.82 ± 83.18	356.33 ± 84.69	338.47 ± 99.07
Scr (*μ*mol/l)	74.32 ± 17.78	76.78 ± 20.48	72.47 ± 17.87	78.19 ± 21.21
eGFR	92.28 ± 22.11	96.26 ± 24.59	98.91 ± 23.99	95.39 ± 24.91
PLT	188.89 ± 52.67	186.15 ± 62.85	198.9 ± 59.95	181.97 ± 63.69

^*∗*^ *P* < 0.05, control vs. other groups; ^#^*P* < 0.05, UAP groups vs. other groups. Data are given as mean ± SD, number (%). ACS, acute coronary syndrome; UAP, unstable angina pectoris; AMI, acute myocardial infarction; BMI, body mass index; ACEIs, angiotensin-converting enzyme inhibitors; ARBs, angiotensin receptor blockers; CCBs, calcium channel blockers; SBP, systolic blood pressure; DBP, diastolic blood pressure; FBS, fasting blood glucose; HbA1c, glycosylated haemoglobin; TC, total cholesterol; LDL-C, low-density lipoprotein cholesterol; HDL-C, high-density lipoprotein cholesterol; TG, triglyceride; SUA, serum uric acid; Scr, serum creatinine; eGFR, estimated glomerular filtration rate; PLT, platelet count.

**Table 2:** PDGF and Ang-1 level of the study population.

Parameter		Control (*n* = 29)	ACS groups		
			Total (*n* = 81)	UAP groups (*n* = 20)	AMI groups (*n* = 61)
PDGF (pg/ml)	Peripheral blood	362.01 ± 292.39	734.08 ± 627.85^*∗*^	614.11 ± 600.39^*∗*^	773.41 ± 636.46^*∗#*^
	Coronary blood		1142.07 ± 837.42^&^	788.93 ± 611.99^&^	1257.87 ± 872.48^#&^

Ang-1 (pg/ml)	Peripheral blood	608.64 ± 237.69	631.98 ± 381.94	553.20 ± 372.96	657.81 ± 384.33
	Coronary blood		128.71 ± 109.94^&^	65.99 ± 40.86^&^	149.27 ± 117.66^&#^

^*∗*^ *P* < 0.01, control vs. other groups; ^#^*P* < 0.05, UAP groups vs. other groups; ^&^*P* < 0.01, peripheral blood level vs. coronary blood level.

## Data Availability

The data used to support the findings of this study were supplied by Xiangming Wang under license. Requests for access to these data should be made to Xiangming Wang [wangxiangming@jsph.org.cn].
